# Multidimensional Single-Nuclei RNA-Seq Reconstruction of Adipose Tissue Reveals Adipocyte Plasticity Underlying Thermogenic Response

**DOI:** 10.3390/cells10113073

**Published:** 2021-11-08

**Authors:** Carlos Alberto Oliveira Biagi, Sarah Santiloni Cury, Cleidson Pádua Alves, Nabil Rabhi, Wilson Araujo Silva, Stephen R. Farmer, Robson Francisco Carvalho, Miguel Luiz Batista

**Affiliations:** 1 Department of Genetics, Ribeirão Preto Medical School, University of São Paulo, Ribeirão Preto 14051-140, Brazil; cbiagijr@gmail.com (C.A.O.B.J.); wilsonjr@usp.br (W.A.S.J.); 2 Center for Cell-Based Therapy (CEPID/FAPESP), National Institute of Science and Technology in Stem Cell and Cell Therapy (INCTC/CNPq), Regional Blood Center of Ribeirão Preto, Ribeirão Preto 14051-140, Brazil; 3 Institute for Cancer Research, IPEC, Guarapuava 85100-000, Brazil; 4 Department of Structural and Functional Biology, Institute of Biosciences, São Paulo State University (UNESP), Botucatu 18618-689, Brazil; santiloni.cury@unesp.br; 5 Department of Translational Genomics, Medical Faculty, University of Cologne, 50923 Cologne, Germany; cleidsonn@gmail.com; 6 Department of Biochemistry, School of Medicine, Boston University, Boston, MA 02215, USA; rabhi@bu.edu (N.R.); sfarmer@bu.edu (S.R.F.); 7 Department of Integrated Biotechnology, University of Mogi das Cruzes, São Paulo 08747-000, Brazil

**Keywords:** mature adipocyte plasticity, adipocyte subpopulations, cellular compartment prediction, transcriptional factors, thermogenic treatment

## Abstract

Adipose tissue has been classified based on its morphology and function as white, brown, or beige/brite. It plays an essential role as a regulator of systemic metabolism through paracrine and endocrine signals. Recently, multiple adipocyte subtypes have been revealed using RNA sequencing technology, going beyond simply defined morphology but also by their cellular origin, adaptation to metabolic stress, and plasticity. Here, we performed an in-depth analysis of publicly available single-nuclei RNAseq from adipose tissue and utilized a workflow template to characterize adipocyte plasticity, heterogeneity, and secretome profiles. The reanalyzed dataset led to the identification of different subtypes of adipocytes including three subpopulations of thermogenic adipocytes, and provided a characterization of distinct transcriptional profiles along the adipocyte trajectory under thermogenic challenges. This study provides a useful resource for further investigations regarding mechanisms related to adipocyte plasticity and trans-differentiation.

## 1. Introduction

The perceived functional complexity of adipose tissue (AT) has changed significantly over the last 30 years since the leptin discovery [1]. The AT is a significant endocrine tissue organized into different depots, which are classified as brown (BAT) or white adipose tissue (WAT) [2]. Mature adipocytes constitute 90% of the AT volume but comprise only 17–33% of total cells. In contrast, the remaining vast majority of cells include a heterogeneous cell population of the stromal vascular fraction (SVF) [3,4]. Mature adipocytes are classified into three distinct types: white, brown, and beige/brite [5]. White adipocytes are responsible for storing triacylglycerides (TGs). The brown adipocytes use lipids to produce heat in part through a UCP1 associated uncoupling of electron transport from ATP production [6]. Beige cells resemble white adipocytes with a shallow basal expression of UCP1, but, like classical brown fat, they respond to cyclic AMP stimulation with high UCP1 expression and respiration rates [7]. These beige cells arise within white fat pads in response to activators such as cold exposure [8], b3-adrenergic receptor (Adrb3) agonists [9], PPARg ligands [5], cancer cachexia [10,11], and exercise training [12,13].

Beige adipocytes have been studied over the last three decades [6]. However, the interest in their physiological function and therapeutic potential to combat obesity has only recently been revisited after discovering the thermogenic response of white adipocytes in adult humans [5,14]. Formation of beige cells in WAT by cold or other stimuli occurs through de novo differentiation of progenitor cells from the perivascular compartment [8] or interconversion of pre-existing white adipocytes [8,15].

Single-cell RNA sequencing (scRNA-Seq) has allowed for the identification of cell-to-cell heterogeneity and plasticity for many different tissues [16,17]. Analysis of adipose tissues at single-cell resolution is challenging. It has several limitations, especially considering the technical limitations of reproducibly isolating the complete adipocyte compartment of the tissue due to the large size and high buoyancy of the adipocytes [18]. Most WAT scRNA-Seq studies to date derive the transcriptomes of the cell types within SVF without providing critical information about the status of the adipocytes in animal models [19,20,21,22,23,24,25,26,27,28,29,30] and humans [22,24,25,31,32,33]. Few studies investigated brown adipocytes [34] or isolated adipocytes from mouse inguinal WAT undergoing browning [35]. Most recently, [36] investigated the complete repertoire of adipose tissue cell types at a single-cell resolution [36]. However, multidimensional studies investigating the mechanisms involved in mature adipocyte plasticity under thermogenic stimuli are still lacking.

Intact cell nuclei have been used to perform single-nuclei RNA-seq (snRNA-seq), overcoming the limitations of isolating the complete adipocyte compartment [37]. The snRNA-seq data of digested adipocytes from inguinal WAT reveals a complex subpopulation of mature adipocytes with distinct genetic signatures [35]. Recently, snRNA-seq analysis of the WAT identified a rare subpopulation of adipocytes in mice that increase in abundance at higher temperatures. This subpopulation regulates the activity of neighboring adipocytes through acetate-mediated modulation of their thermogenic capacity [38].

Here, we show an effective suitable pipeline able to reconstruct the mature adipocyte heterogeneity of the thermogenic response at the single-nuclei resolution. Our analyses generated a comprehensive and expansive cellular atlas presenting three thermogenic adipocyte subpopulations, followed by additional information on the metabolic pathways, the plasticity of individual subpopulations, and transcription factors possibly involved in beige remodeling of WAT. Also, we characterized specific cell surface markers and the secretome for each adipocyte subpopulation. The detailed snRNA-Seq analysis presented herein of the transcriptional changes in WAT adipocytes under thermogenic challenge provides insight into the molecular mechanisms driving adipocyte plasticity.

## 2. Materials and Methods

### 2.1. Single-Cell RNA-Seq Data

The scRNA-seq data from mature adipocytes under cold-challenge and B3-adrenergic agonist stimulation were acquired from the Gene Expression Omnibus (GEO) database under the series number GSE133486 [35], which contains six data of mouse SVF and 10 data of mouse adipose nuclei generated using Drop-Seq and 3’ V3 chemistry kit on Chromium Single-cell controller (10× Genomics), respectively.

### 2.2. Data Pre-Processing

The filtered feature-barcode matrix was used in the following analysis. All additional analysis were performed using Seurat v3 [39,40] R package. First, to reproduce the results obtained by Rajbhandari et al. [35], the same procedures described in the paper were used. For the single nuclei data reanalysis, only data of mouse adipose nuclei wild-type mice underwent cold-challenge and B3-adrenergic agonist stimulation were used. Nuclei with less than 200 and more than 3000 genes detected, with more than a 10% percentage of mitochondrial genes, and with P condition were excluded from the analysis. We assigned scores for S and G2/M cell cycle phases based on previously defined gene sets [41] using the CellCycleScoring function for clustering of all cells. Regularized negative binomial regression was used to normalize UMI count data using the sctransform workflow [42], regressing out against the number of UMIs per cell, S phase score, and G2/M phase score. Scaled data was used as an input into PCA based on variable genes. Clusters were identified using shared nearest neighbor (SNN)-based clustering based on the first 26 PCs (corresponds to a PCA cumulative proportion greater than 80%) and resolution = 1. The same principal components were used to generate the *t*-SNE projection, which was developed with a maximum of 2000 iterations.

### 2.3. Optimal Number of Clusters

To find the optimal number of clusters, the SCCAF was used with an accuracy threshold of 80%. The clustering calculated previously was used as the initial clusters, and the h5ad file used as input was generated using the SeuratToH5ad function. The optimize, skip-assessment, produce-rounds-summary, and optimisation-plots-output parameters were used in this first step to use the sccaf command to round optimization. Then the sccaf-assess command was used to determine the round to be used as a final result by through the observation of accuracies for each round on multiple iterations. For this step the default parameters were used using 20 iterations. Finally, the last step was to generate a plot used to compare the accuracy between different rounds. For this purpose, the sccaf-assess-merger command was employed using the results from step one and two.

### 2.4. Cell-Type Classification

To classify the cell types, Metacell [43] was used with the default parameters. Firstly, we used some initial markers such as *Adrb3* for Adipocyte, *Pecam* for Endothelial, *Ptprc*, and *Cd19* for Immune and *Cd34*, and *Pdgfra* for Progenitor cell types. Based on these markers and cell types, the tool returned a list of new markers that can separate these cell types. To select these new markers, we performed isolation of each cell type group to check each marker’s expression. The markers that had an expression in the given group greater than 90% of the quantile were considered markers for the respective group. The markers were: *Acsl1*, *Plin4*, *Mlxipl*, *Pck1*, and *Adrb3* for Adipocyte, *Btnl9*, *Ushbp1*, *Egfl7*, *Mcf2l* and *Ptprb* for Endothelial, *Zeb2*, *Trps1*, *Runx1*, *Ptprc*, and *Adap2* for Immune, *Dcn*, *Celf2*, *Meg3*, *Col1a2* and *Col3a1* for Progenitor cell types. So, based on these markers, the tool was able to classify the cell types.

### 2.5. Optimal Mature Adipocyte Subclusters

The mature adipocyte cluster was subsetted using an optimal number of clusters, while the SCCAF (described above) was used with an accuracy threshold of 80%. RT (Ad3 e Ad4), Cold (Ad1 e Ad2), CL (Ad1 e Ad5); adipocyte clusters of cells (Ad1, Ad2, Ad3, Ad4 and Ad5).

### 2.6. Differential Expression and Enrichment Analysis

Differentially expressed genes between the different conditions each cluster/cell type were identified using FDR < 0.05 and/or |avg_logFC| > 0.25. Functional enrichment analysis was performed using the Enrichr tool v3.0 R package [44,45]. For better visualization of the data, the adaptively-thresholded Low Rank approximation (ALRA) [46] imputation method and Nebulosa v1.2.0 (Kernel Gene-Weighted Density Estimation) [47] R package was used.

### 2.7. Transcriptome-Based Secretome Analysis

The differentially expressed genes in the mature adipocyte subclusters (Ad1, Ad2, Ad3, Ad4, and Ad5) were filtered for genes encoding secreted proteins based on a pipeline of four databases and tools. UniProtKB [48] annotation of subcellular localization was accessed to select proteins classified as “Secreted” and Gene Ontology (GO) [49] annotation of the cellular component was used for selection of “Extracellular” proteins. To confirm those results, the combined lists of proteins generated by UniprotKB and GO were analyzed using the algorithms SignalP 5.0 [50], SecretomeP 2.0 [51]. SignalP server [52] was used to identify classical secretory proteins (presenting signal peptide considering the D-value > 0.45). Proteins without signal peptide were evaluated in the SecretomeP 2.0 server to determine non-classical secreted proteins, using the cutoff for a neural network (NN) score > 0.6. The same strategy was used for clusters of adipocytes highly expressing *Ucp1* compared to adipocytes with lower levels of *Ucp1*. Each condition’s predicted secretome was visualized using a heatmap plot using Morpheus software [53]. We also verified the secretion via exosomes by accessing the Exocarta V.5 database (http://www.exocarta.org/, accessed on 28 October 2021) [54].

### 2.8. Membranome Prediction

The differentially expressed genes in the mature adipocyte subclusters (Ad1, Ad3, Ad4, and Ad5) were filtered for genes encoding cell membrane proteins. UniprotKB and GO were also used for the membranome annotation filtering in the proteins classified at “cell membrane” and “plasma membrane”, respectively. The list generated was confirmed using the TMHMM 2.0 [55] algorithm, selecting only the proteins with a predicted number of transmembrane helices (PredHel) greater than 1. This prediction analysis classifies transmembrane proteins without discriminating if the protein is located on plasma or vesicles or organelles membranes. Thus, we manually reviewed the literature of our selected top five genes of interest to confirm which membrane they belong to. The same strategy was used for subclusters of adipocytes highly expressing *Ucp1* compared to adipocytes with lower levels of *Ucp1*.

### 2.9. Cell-Cell Interaction

We used the computational framework CellPhoneDB v2.1.7 in Python to predict cell-cell communication using its repository of curated ligand-receptor interactions for single-cell transcriptomic data [56,57]. We used the default setting to select the statistically relevant interaction (*p*-value < 0.05) between the mature adipocyte subclusters belonging to RT group (Ad3 vs. Ad4) and the CL group (Ad1 vs. Ad5).

### 2.10. Pseudotime Analysis

To analyze the trajectory development of adipocyte clusters, an unsupervised pseudo temporal analysis was performed using Monocle2 v2.20.0 [58,59,60] R package. The Seurat object with cluster information was extracted and converted to a Monocle2 CellDataSet. Monocle2 uses DDRTree, a reversed graph embedding algorithm to predict biological trajectories to reduce the high-dimensional scRNA-seq data space and predict how cells progress through a given biological process based on global gene expression levels. Monocle2 offers ideal unsupervised pseudotime analysis for this study, as it indicates branch points and trajectory states without cell fate input information. Following size factor and dispersion estimates, trajectory ordering genes were called by testing the differential expression of genes expressed with min_expr = 0.1 in ≥10 cells against the five clusters of adipocytes, selecting the genes that have qval < 0.01. Data dimensionality was reduced using the reduceDimension function with max_components set to 2 and reduction_method set to DDRTree. The cells were ordered according to the state that represents the initial condition (state 1). DEGs across pseudotime were determined using the differentialGeneTest function filtering by qval < 0.01. Resultant genes were ordered by q value, and the top-500 genes changing in pseudotime were visualized using the plot_pseudotime_heatmap function.

### 2.11. Transcription Factors (tfs) Enrichment Analysis and Protein-Protein Interaction (PPI)

We used the eXpression2Kinases (X2K) v. 2.0 [61,62] workflow to identify the upstream TFs of the DEGs within subclusters with high and low gene expression of *Ucp1*. We selected the enriched TFs (*p*-value < 0.05) to construct the PPI network with their targeted genes found as DEGs in the adipose single-cells expressing or not *Ucp1* in cluster Ad1. PPI networks were conducted using STRING v.11.0 (https://string-db.org/, accessed on 28 October 2021). Only medium confidence interactions were included (interaction score of at least 0.4), and the disconnected nodes were omitted in the network. Visualization and data annotation of PPI networks were constructed using Cytoscape v3.7.2 [63]. The CytoNCA plugin [64] was used to calculate the betweenness centrality values of each node.

### 2.12. Data Representation

Heat-scatter plot was generated using Morpheus [53] (https://software.broadinstitute.org/morpheus, accessed on 28 October 2021). Venn diagrams were plotted using the web server jvenn [65].

### 2.13. Data and Code Availability

The accession number for the single-nuclei sequencing data from Rajbhandari et al. (2019) [35] reported in this paper is GEO: GSE133486. All analysis code is available on GitHub at https://github.com/cbiagii/snRNAseq_adipocyte, accessed on 28 October 2021.

## 3. Results

### 3.1. Multidimensional snRNA-Seq Reconstruction Reveals Distinct Adipocyte Subpopulations Derived from Mouse iWAT

**Figure 1:**
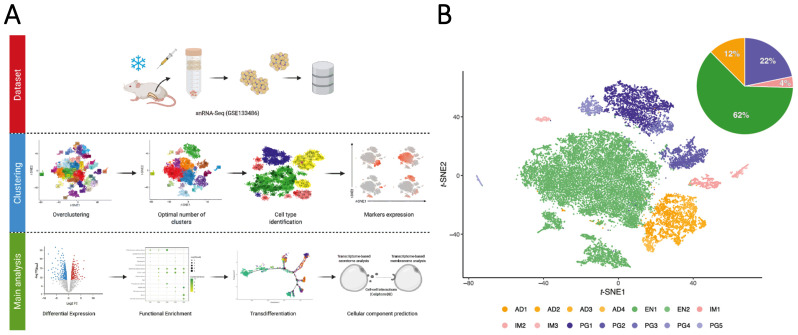

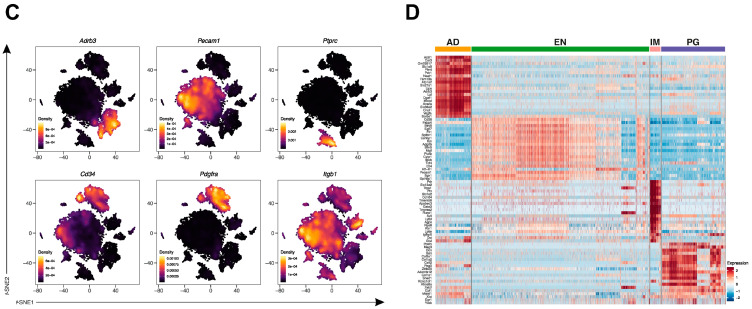

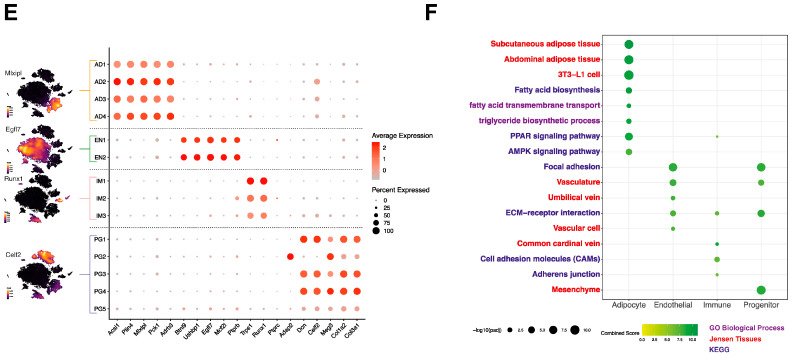
Adipose Single-Nuclei Workflow Template identifies different cell populations in adipose tissue. (**A**) Workflow overview showing the three main steps to process the template. The first step corresponds to obtaining the dataset (in this paper public data was used with accession number GSE133486). The next step is to cluster the data that includes the over clustering, finding the optimal number of clusters, cell type identification, and marker expression. The last step is related to the principal analysis that includes differential expression, functional enrichment, trans-differentiation, and cell component prediction. (Created with BioRender.com) (**B**) *t*-SNE plot of 28,820 single nuclei cells distributed by 14 clusters in four cell types: adipocyte (AD), endothelial (EN), immune (IM), and progenitor (PG). Pie charts show the corresponding percentage. (**C**) Nebulosa representing the unsupervised clustering of six canonical cell type markers for each cluster. *Pdgfra*, *Itgb1*, and *Cd34* (for adipocyte progenitors and stem cells), *Ptprc* (for immune cells), *Pecam1* (for endothelial cells), and Adrb3 (for mature primary adipocytes). (**D**) Gene-expression heatmap of the top 20 DEGs in each defined cell type compared to all others. Genes are represented in rows and cell clusters in columns. (**E**) Gene-expression dot plot of select top five DEGs for each defined cell type. Rows depict clusters, while columns depict genes. The intensity of any given point indicates average expression, while its size represents the proportion of cells expressing a particular gene. (**F**) Selected top categories from ORA analysis of DEGs from the four cell types identified. The intensity of the color in the dotplot indicates the enrichment significance by the combined score. Circle sizes correspond to the -log10 adjusted *p*-value (padj). Gene set names are colored according to the GO biological process (purple), Jensen tissues (red), and Kyoto Encyclopedia of Genes and Genomes (KEGG, blue).

We sought to characterize the transcriptional profiles of adipocytes by reanalyzing single-nuclei RNA sequencing data of isolated primary adipocytes responding to different thermogenic stimuli: 4 °C challenge for four days (Cold) and CL-treatment, 1 mg/kg/day for four days (CL). We selected the experimental challenges to increase the chance of detecting nuclei of mature primary adipocyte populations. For cells obtained from iWAT samples (from now, referred to as fat-cake), the *t*-SNE plots revealed 17 distinct nuclei clusters at different experimental conditions (Figure S1A). Those distinct clusters were subjected to a workflow template, depicted in Figure 1A. The first step of our pipeline was to subject raw data to over-clusterization (SCCAF), followed by the identification and classification of the cellular heterogeneity (MetaCell) (Figure S1B). We annotated the clusters of nuclei using marker genes (described in detail in the STAR Methods), which resulted in the identification of four groups of cell clusters: progenitor cells (PG), immune cells (IM), endothelial cells (EN), and adipocytes (AD1) (Figure 1B). The proportion of cell types (average) per individual was 22% for progenitors, 10% for immune cells, 55% for endothelial cells, and 13% for adipocytes (Figure S1B). An accuracy threshold of 80% was used for cluster optimization (Figure S1C), and round 3 was chosen as the best round based on the accuracy and cross-validation test (Figure S1D). The distribution was similar after SCCAF over-clusterization: 22% for adipocyte progenitors and stem cells (PG1–PG5), 4% for immune cells (IM1–IM3) and 62% for endothelial cells (EN1–EN2), and 12% for adipocytes (AD1–AD4) (Figure 1B). Thus, unsupervised clustering of the single-nuclei transcriptional profiles identified four adipocyte subsets in the iWAT fat-cake. The following canonical cell type markers were upregulated in these clusters: *Pdgfra*, *Itgb1*, and *Cd34* (for adipocyte progenitors and stem cells), *Ptprc* (for immune cells), *Pecam1* (for endothelial cells), and Adrb3 (for adipocytes) (Figure 1C).

After MetaCell analysis, a list of genes used to define each of the different clusters is presented in Figure S1E and Table S1. The expression profile of the top 20 cell-type-specific DEGs is shown in Figure 1D and Table S2. Unsupervised analysis of DEGs identified four significant adipocyte populations (i.e., expressing *Acsl1*, *Plin4*, *Mlxipl*, *Pck1*, and *Adrb3*), a population of endothelial cells (*Btnl9*, *Ushdp1*, *Egfl7*, *Ncf2l*, and *Ptprb*), adipocyte progenitors and stem cells (*Dcn*, *Celf2*, *Meg3*, *Col1a2*, and *Col3a1*), and immune cells (*Trps1*, *Runx1*, *Ptprc*, and *Adap2*) (Figure 1E). Adipocyte clusters enriched genes associated with subcutaneous adipose tissue and PPAR signaling. Endothelial cells enriched genes are related to focal adhesion and vasculature, immune cells are significantly enriched with cell adhesion molecules genes, and adipocyte progenitors and stem cells are enriched for mesenchyme cells and myofibroblasts genes (Figure 1F and Figure S1F and Tables S3 and S4). These transcriptional differences may underlie distinct functional characteristics of the different cell types identified in the single-nuclei RNA-Seq reanalysis pipeline.

### 3.2. Reclustering of Adipocyte Clusters Reveals Two Distinct Mature Adipocyte Populations at Room Temperature

**Figure 2:**
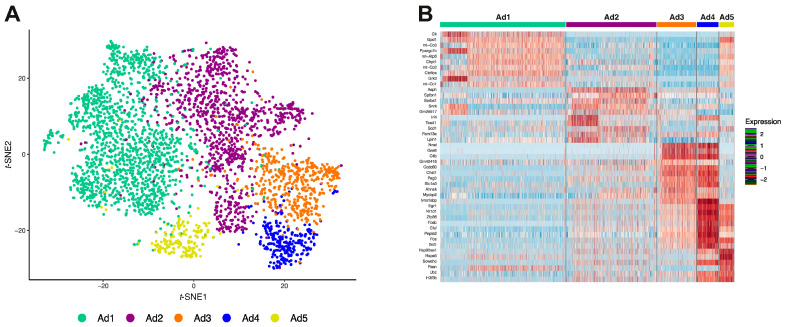

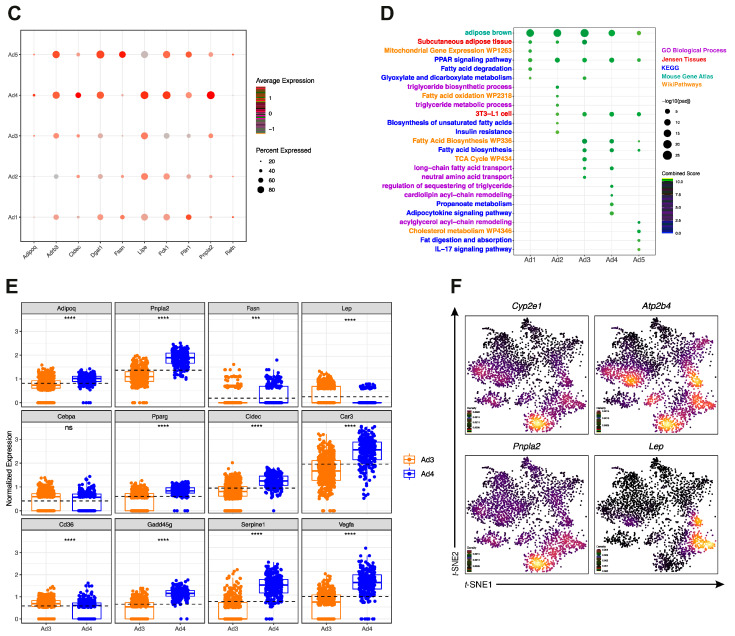
Multidimensional workflow template identifies two distinct mature adipocyte subclusters at RT. (**A**) Reclustering of adipocytes nuclei using SCCAF identified 5 putative adipocyte subclusters from “fat cake” iWAT nuclei data (Ad1 = 1539 nuclei; Ad2 = 1106 nuclei; Ad3 = 477 nuclei; Ad4 = 265 nuclei and Ad5 = 181 nuclei). (**B**) Gene-expression heatmap of the top 10 most DEGs in each adipocyte subcluster compared to all others. Genes are represented in rows and cell clusters in columns. (**C**) Gene-expression dot plot of the canonical adipocyte markers for each adipocyte subcluster. Rows depict clusters, while columns depict genes. The intensity of any given point indicates average expression, while its size represents the proportion of cells expressing a particular gene. (**D**) Selected top categories from ORA analysis of DEGs from the five adipocyte clusters. The intensity of the color in the dotplot indicates the enrichment significance by the combined score. Circle sizes correspond to the -log10 adjusted *p*-value (padj). Gene set names are colored according to the GO biological process (purple), Jensen tissues (red), Kyoto Encyclopedia of Genes and Genomes (KEGG, blue), Mouse Gene atlas (green), and WikiPathways (orange). (**E**) Scatter-box plot representing normalized gene expression of 12 marker genes in the two adipocyte clusters from RT (Ad3 and Ad4 subclusters). At the top of each box is the significance of the comparison between Ad3 and Ad4 subclusters. ns, non-statistical significance, *** *p* < 0.005, **** *p* < 0.0001. (**F**) Nebulosa expression plots representing four markers (*Cyp2e1*, *Atp2b4*, *Pnpla2* and *Lep*).

To gain insight into the molecular differences between adipocyte subpopulations, we first verified the accuracy threshold of 80% that was used to cluster optimization (Figure S2A), and round 3 was chosen as the best round based on the accuracy and cross-validation test (Figure S2B). Next, we applied unsupervised over-clustering (SCCAF) to partition all 3568 adipocytes nuclei that were identified using *t*-SNE. Interestingly, it identified five distinct adipocyte subpopulations (Ad1–Ad5, Figure 2A), each having a particular DEG pattern, with a slight exception for the Ad3 and Ad4 subpopulations (Figure 2B and Table S5). The canonical adipocyte markers *Dgat1*, *Plin1*, *Lipe*, *Cidec*, were expressed in all adipocyte subpopulations (Figure 2C and Figure S2C), albeit at varying levels. We found 571 DEGs in Ad1 (362 up and 209 down-regulated); 281 DEGs in Ad2 (118 up- and 163 down-regulated); 294 DEGs in Ad3 (70 up- and 224 down-regulated); 412 DEGs in Ad4 (353 up- and 59 down-regulated); and 160 DEGs in Ad5 (156 up- and four down-regulated) (Figure S2D and Table S6). We applied functional enrichment analysis of the five adipocyte subpopulations (Ad) (Figure 2D and Figure S2E, Tables S7 and S8). The DEGs capture significant aspects of heterogeneity in distinct adipocyte subpopulations. Such differences were reflected in mitochondria gene expression and fatty acid degradation for Ad1, triglycerides biosynthetic process for Ad2, and ATP biosynthetic process for Ad1 and 2, TCA cycle and acetyl-CoA metabolic process, and regulation of cell differentiation for Ad3, regulation of sequestering triglycerides, and long-chain fatty acid transport, and adipocytokine signaling pathways for Ad4 and cholesterol metabolism for Ad5. Interestingly, fatty acid biosynthetic processes and long-chain fatty acid transport, and white adipose tissue (mouse-genes-atlas) were predominantly enriched in the Ad3 and Ad4 subpopulations. Interestingly, using the database Jensen tissues, we observed that the Ad3 subpopulation was the only one significantly enriched for the “Preadipocyte cell line” term (Figure S2E), suggesting a “preadipocyte-like” expression profile specifically found in the Ad3 (*Cfd*, *Fabp4*, *Gpd1*, and *Lpl*). Ad3 and Ad4 adipocyte subpopulations appeared to represent classical adipocytes, and they expressed genes associated with WAT, *Cidec*, *Pnpla*, and *Adipoq* (Figure S2E).

Ad3 and Ad4 subpopulations correspond to adipocytes present in fat cake of iWAT of non-treated mice (Control, RT) (Figure 2A). A comparison of adipocyte canonic markers revealed that Ad4 expresses higher levels of *Adipoq*, *Plpna2*, *Fasn*, *Pparg*, *Cidec*, *Car3*, *and Gadd45g* than Ad3 at room temperature (Figure 2E) suggesting that Ad4 more so than Ad3 consists of “classic” adipocytes. Interestingly, leptin is more highly expressed in Ad3 than in the Ad4 subpopulation (Figure 2E). Interestingly, higher expression of *Cyp2e1 and Atp2b4* were detected predominantly in Ad4 (Figure 2F). Recently, *Cyp2e1 and Atp2b4* were shown to be restricted to the mature adipocyte fraction in BAT and WAT [66], and such adipocyte populations have been identified as controls for the thermogenic function of other adipocytes [38].

To gain additional insight into Ad3 and Ad4 subsets, we performed an analysis of the secretome and membranome using gene expression profiles (DEGs). 29 genes predicted to encode membrane proteins were upregulated exclusively in Ad4, while only two genes were upregulated in Ad3 (*Ntrk2* and *Atp1a2*), and nine other membrane genes were differentially expressed in both subpopulations, showing the top five highly expressed genes, *Irs2*, *Abca1*, *Mrap*, *Irs1*, and *Adrb3*, which could potentially be used as Ad4 subpopulation markers (Figure S2G and Table S9). Regarding the secreted proteins, we found that Ad3 overexpresses 17.4% of genes that encode secretory proteins, while Ad4 overexpresses only 8.2% (Figure S2F). The top five exclusively expressed in Ad3 (*Gpx3*, *Col1a2*, and *Spon1*) and Ad4 (*Vegfa*, *Serpine1*, *Angptl4*, *Hspa8*, and *Cesd1*) subpopulation are detailed in Figure 2G.

Characterization of the secretory proteins and components of the cell membrane permitted a prediction of cell-cell interactions via ligands and possible receptors (i.e., interactome) (Figure S2H). Ad3 and Ad4 showed increased interaction through collagens (produced by Ad3) and integrin (Ad4) and decreased interaction through NOTCH1 (Ad3) with JAG1 (Ad4) and COL5A1 (Ad3) and integrin complex (Ad4). Ad4 interacts with Ad3 by producing the ADIPOQ ligand interacting with the CLEC2D receptor in Ad3.

### 3.3. Identification of a Unique Adipocyte Thermogenic Subpopulation Corresponding to Both Cold and CL-Treatment

**Figure 3:**
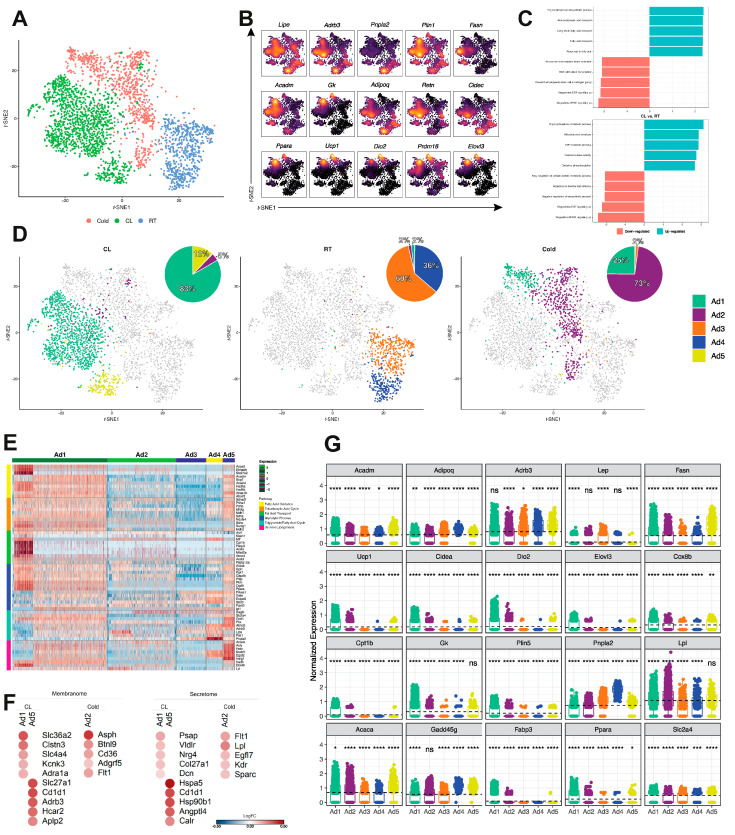

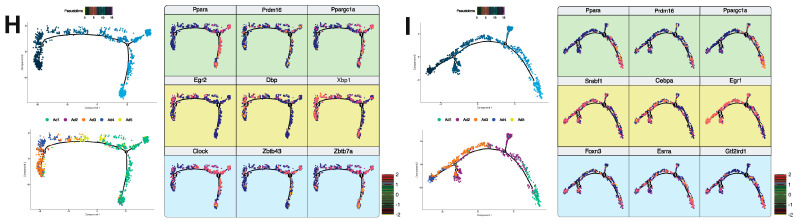
CL treatment and cold-challenge reveals a single thermogenic subpopulation. (**A**) *t*-SNE representation showing a subset of mature adipocyte nuclei subclusters classified according to each treatment (Cold = 892 cells: CL = 1433 cells and RT = 702 cells). (**B**) Nebulosa expression plots of canonical mature adipocyte and thermogenic genes. (**C**) Gene Set Enrichment Analysis (GSEA) comparing Cold vs. RT and CL vs. RT. Rows depict enriched pathways, while on the y-axis, the Normalized Enrichment Score (NES). Positive NES represents an upregulated pathway, and negative NES represents down-regulated pathways. (**D**) CL, RT, and Cold shown subset of adipocyte nuclei subclusters classified according to each treatment integrated in *t*-SNE plot (CL = 1443 cells; RT = 702 cells and Cold = 892 cells). Pie charts show the corresponding percentage. (**E**) Heat map showing the expression of main genes related to fatty acid oxidation, tricarboxylic acid cycle, fatty acid transport, glycolytic process, triglyceride/fatty acid cycle, and de novo lipogenesis in the five adipocytes subclusters. (**F**) Heat-scatter plot representing top five upregulated membranome components specifically identified in Ad1 subcluster and the top five exclusive membranome of Ad5 subcluster from CL subset comparison. The top five membrane genes from subcluster Ad2 (Cold) are demonstrated separately (left). A heat-scatter plot representing the top five upregulated secretome components specifically identified in Ad1 subcluster and the top five exclusive secretome of Ad5 subcluster. The top five secretome genes from subcluster Ad2 (Cold) are demonstrated separately (right). The color of the circles corresponds to the average logFC. FC: fold change (**G**) Scatter-box plot representing normalized gene expression of 20 markers of canonical adipocyte and thermogenic genes in the five adipocyte subclusters. At the top of each box is the significance. ns, non-statistical significance, * *p* < 0.05, ** *p* < 0.01, *** *p* < 0.005, **** *p* < 0.0001. (**H**) Monocle-generated plots presenting pseudotime ordering and differentiation trajectory of CL and RT conditions. The trajectory suggests a transition between Ad3-Ad4-Ad5-Ad1. Green background represents the three main thermogenic TFs (classic). The yellow background represents the earlier expressed TFs in the trajectory, and the blue background represents the later expressed TFs in the trajectory. The characterized genes are DEGs throughout the trajectory. (**I**) Monocle-generated plots presenting pseudotime ordering and differentiation trajectory of Cold and RT conditions. The trajectory suggests a transition between Ad4-Ad3-Ad2-Ad1. Green background represents the three main thermogenic TFs (classic). The yellow background represents the earlier expressed TFs in the trajectory, and the blue background represents the later expressed TFs in the trajectory. The characterized genes are DEGs throughout the trajectory.

To further identify the adipocyte subpopulation with thermogenic transcriptome signature, we performed unbiased aggregated clustering of the processed data for each of the experimental conditions, i.e., Cold, CL and RT, as a *t*-SNE-plot (Figure 3A). The aggregated cluster represents 3027 adipocyte nuclei. Figure 3B shows the gene expression of the selected (supervised) adipocyte and thermogenic markers. For these data, it is interesting to note that the profile of gene distribution of *Adipoq*, *Retn*, *Cidec*, *and Fasn* (canonical adipocyte markers) has almost no overlap with thermogenic genes such as *Ppara*, *Ucp1*, *Dio2*, *Prdm16*, *Elovl3*. Figure S3A and Table S10 highlight the DEGs related to cold-challenge (Cold) and CL treatment. The top 5 DEGs for cold-challenge were *Acacb*, *Acss2*, *mt-Co2*, *Macf1*, and *Gm26917*, while for CL were *Acsl1*, *mt-Co3*, *mt-Co2*, *mt-Atp6*, *Fasn.* Once we determined that the two treatments (Cold and CL) have different gene expression profiles, we performed functional enrichment analysis (Figure 3C and Figure S3B, Table S11–S13), using enriched genes in each cluster based on different experimental conditions. This analysis revealed that different adipocyte subclusters express distinct genes corresponding to the experimental conditions. For example, cold-induced subclusters demonstrated critical organophosphate biosynthetic processes and fatty acid transport. At the same time, CL showed significant aspects of organophosphate metabolic processes, oxidative phosphorylation, and ATP metabolic processes. The down-regulated genes reveal negative regulation for biosynthetic processes and response to mechanical stimuli for both experimental conditions.

Since we have observed that RT, CL, and cold presents distinct frequencies in the adipocyte subpopulations, we analyzed the distribution of all adipocytes highlighted according to each different experimental condition (Figure 3D). Integrated analysis for adipocyte nuclei of CL, RT, and cold treatments revealed five subclusters, with Ad1 (83%) and Ad5 (12%) mainly from CL, Ad3 (60%) and Ad4 (36%) derived primarily from RT, and Ad2 (73%) and Ad1 (25%) mainly from cold. Interestingly, Ad1 contained adipocyte nuclei from both CL and Cold conditions and was more prevalent in Cl than other subpopulations. Whereas CL and Cold both exhibited unique expression patterns that reflected their functional commitments, CL showed significant functional enrichments mainly related to fatty acid degradation and transportation (Figure S3B). Unsurprisingly, the Ad1 subpopulation was most heavily involved in fatty acid oxidation, TAC, and fatty acid transport (Figure 3E and Figure S3C). The latter seems to be very specific to Ad1. The expression profiles of genes corresponding to glycolytic process and triglycerides/fatty acid cycle, however, were not so distinguished between Ad1 *versus* Ad5 subpopulation (Figure 3E), despite the fact the Ad5 showed relatively higher expression of most of the metabolism-related genes, in particular, triglycerides/fatty acid processes and de novo lipogenesis; this is indicative of a high energy demand for Ad5 subpopulation.

Identification of three distinct thermogenic adipocyte subpopulations led us to predict corresponding secretome and membranome (Figure 3F and Figure S3D–G, Table S14). In Ad1 and Ad5, 7.2% and 9%, respectively, of upregulated genes represent membrane encoding proteins (Figure S3E). Two genes were exclusive to both Ad1 and Ad5 clusters (Figure S3D), while 23 plasma membrane genes were upregulated exclusively in Ad1 and 12 genes in Ad5; the top five expressed genes in each subpopulation are *Slc36a2*, *Clstn3*, *Slc4a4*, *Kcnk3*, *Adra1a* for Ad1, and *Slc27a1*, *Cd1d1*, *Adrb3*, *Hcar2*, *Aplp2* for Ad5 (Figure 3F). The Ad1 subcluster was found to overexpress 3.3% of potential secreted proteins and Ad5 overexpressed 5.8%. The secretory genes exclusively expressed in Ad1 and Ad5 subpopulations were Ad1: *Psap*, *Vldlr*, *Nrg4*, *Col27a1*, *Dcn*; Ad5: *Hpsa5*, *Cd1d1*, *Hsp90b1*, *Angptl4*, *Calr* (Figure 3F). The complete list of the predicted secreted and membrane proteins in Ad1 and Ad5 subpopulations are presented in Figure S3F,G. Ad2 shows 12.7% of upregulated genes are membrane encoding proteins (*Asph*, *Btnl9*, *Cd36*, *Adgrf5*, and *Flt1*) and 9.3% are secreted proteins (*Flt1*, *Lpl*, *Egfl7*, *Kdr*, and *Sparc*). VEGFA was the main interacting molecule between Ad1 and Ad5 subclusters (Figure S3H).

The gene expression profile (normalized) of the adipocyte subpopulations shows that the classic thermogenic markers, *Ucp1*, *Cidea*, *Dio2*, *Elovl3*, *Cpt1b*, and *Plin5*, are differentially expressed in all clusters, but particularly in Ad1 (Figure 3G). Other genes, such as *Acadm*, *Cox8b*, and *Gk* seem to be closely related to CL subclusters (Ad1 and Ad5). *Fasn* and *Acaca* showed a higher level of gene expression in Ad5 than all other subpopulations, which may indicate a specific profile in this adipocyte phenotype.

Since reanalysis of the snRNA-seq dataset simultaneously profiles adipocytes under both cold and CL-treatment, we hypothesized that their plasticity could be traced in vivo by mapping a developmental trajectory between distinct adipocyte subpopulations. As two significant subpopulations were resolved in the RT nuclei dataset (Ad3, Ad4) from iWAT (Figure 3D), we established this condition as the starting point for the analysis through the Monocle. Pseudotime study mapped a distinct trajectory of RT adipocytes responding to thermogenic challenge into different cellular states, ranging over modifications to late thermogenic trans-differentiation (Figure 3H,I). This identified a branched trajectory connecting the adipocyte subpopulations with two branches representing the adipocytes’ specification into distinct subpopulations. We focused on the thermogenic branch 1 to separate the nuclei into late trans-differentiation (mostly Ad1), and in branch 2, related to early trans-differentiation (mostly Ad3) (Figure S3I,J).

In the CL subpopulations (Ad1, Ad5), adipocytes lost expression of transcription factors (TF) *Egr2*, *Dpb*, and *Xbp1*, as well as of genes *Nnat*, *Plppr3*, and *Chst1* (Figure S3I). A progressive gain of expression is shown for TFs, *Clock*, *Zbtb43*, and *Zbtb7a*, whereas “classic” thermogenic TFs *Ppara*, *Prdm16*, and *Ppargc1a* are expressed along the entire trajectory analysis. *Ucp1* was expressed in late trans-differentiation thermogenic adipocytes, followed by *Ppara* and *Dio2* (Figure S3I and Table S15). The cold-challenged adipocytes (Ad1 and Ad2) lost expression of *Egr1*, *Cebpa*, and *Srebf1* and progressively gained expression of TFs *Foxn3*, *Essra*, and *Gtf2ird1* (Figure 3I). Brown adipocyte markers were also expressed in late trans-differentiation thermogenic adipocytes (Figure S3J and Table S16).

### 3.4. Ad1-Ucp1^High^ Subpopulation Shows a “Classical” Thermogenic Profile

**Figure 4:**
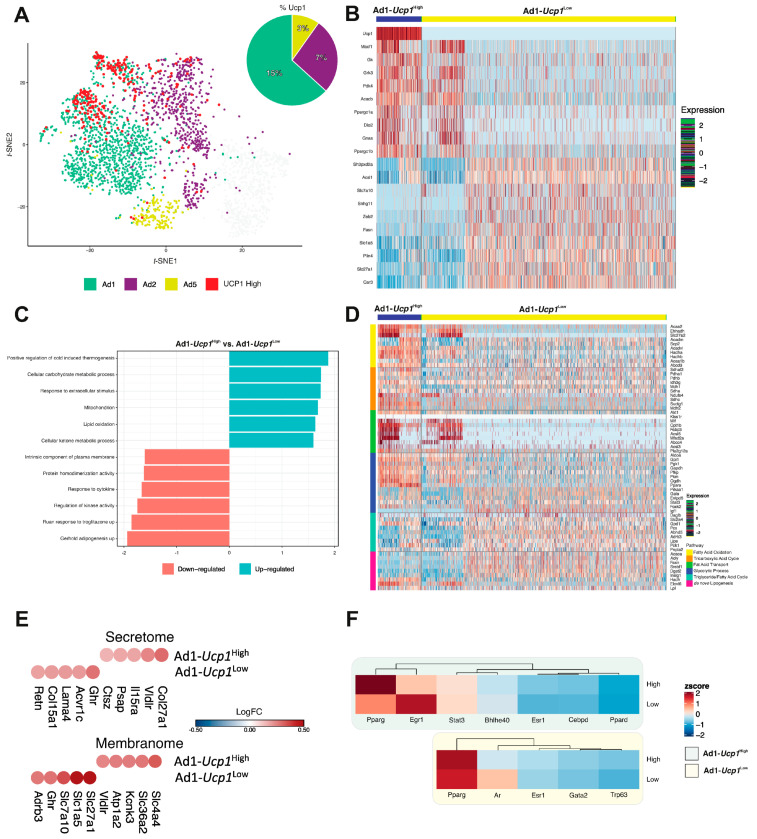

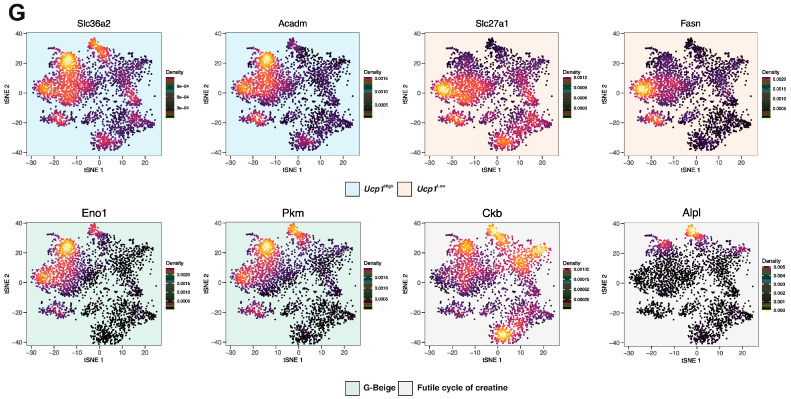
Ad1-*Ucp1*^High^ subpopulation shows a “classical” thermogenic profile whereas Ad1-*Ucp1*^Low^ subpopulation suggest a potential UCP1- independent pathways activation. (**A**) *t*-SNE representation showing Ad1, Ad2 and Ad5 mature adipocyte nuclei subclusters. The red dots represent the cells that have higher expression levels of *Ucp1*. Pie charts show the corresponding percentage of high *Ucp1* in each of Ad1, Ad2 and Ad5 subclusters. (**B**) Gene-expression heatmap of the top 10 most DEGs in Ad1-*Ucp1*^High^ vs. Ad1-*Ucp1*^Low^ comparison. Genes are represented in rows and cell clusters in columns. (**C**) Gene Set Enrichment Analysis (GSEA) comparing Ad1-*Ucp1*^High^ vs. Ad1-*Ucp1*^Low^. Rows depict enriched pathways, while in y-axis the Normalized Enrichment Score (NES). A positive NES represents an upregulated pathway and negative NES represents down-regulated pathways. (**D**) Heat map showing the expression of main genes related to fatty acid oxidation, tricarboxylic acid cycle, fatty acid transport, glycolytic process, triglyceride/fatty acid cycle, and de novo lipogenesis in the Ad1-*Ucp1*^High^ and Ad1-*Ucp1*^Low^. (**E**) Heat-scatter plot representing the top five upregulated secretome components identified in Ad1-*Ucp1*^High^ and Ad1-*Ucp1*^Low^ (upper plot). Heat-scatter plot representing the top five upregulated membranome components identified in Ad1-*Ucp1*^High^ and Ad1-*Ucp1*^Low^ (bottom plot). The color of the circles corresponds to the average logFC. (**F**) Heatmap showing the average expression for seven main TFs in Ad1-*Ucp1*^High^ cells (green background) and five main TFs in Ad1-*Ucp1*^Low^ cells (yellow background). These TFs are related to the DEGs from each comparison through the Transcription Factor Enrichment Analysis (TFEA) using the X2K database. (**G**) Nebulosa expression plots representing four positive/negative markers for *Ucp1* (*Slc36a2*, *Acadm*, *Slc27a1* and *Fasn*) (on top). Nebulosa expression for G-Beige (*Eno1* and *Pkm*) and Futile cycle of creatine (*Ckb* and *Alpl*) (bottom).

Once the Ad1 subpopulation was shown to express a clear thermogenic signature in response to both Cold- and CL-challenge, the next step was to evaluate Ad1 nuclei that express high levels of UCP1 and compare them with those expressing low levels of UCP1 (details in STAR Method). Figure 4A presents the *t*-SNE representation of adipocyte subpopulations Ad1, Ad2, and Ad5 highlighting nuclei with high expression of *Ucp1* (from hereafter, Ad1 will be named Ad1-*Ucp1*^High^ and *Ucp1*^Low^). The thermogenic genes *Ppara*, *Dio2*, *Prdm16*, *Elovl3*, *Cox8b* are more prevalent in the Ad1 subpopulation (Figure S4A). The top-5 DEGs in Ad1-*Ucp1*^High^ nuclei are *Macf1*, *Gk*, *Grk3*, *Pdk4*, and *Acacb* (Figure 4B and Figure S4B and Tables S17 and S18). Interestingly, it should be noted that a few adipocyte nuclei belonging to Ad1-*Ucp1*^Low^ have a gene signature similar to Ad1-*Ucp1*^High^.

To gain mechanistic insight into gene lists, we applied enrichment analysis to evaluate the pathways enriched in a gene list of Ad1 (*Ucp1*^High^ and *Ucp1*^Low^). As expected, for Ad1-*Ucp1*^High^, pathways related to positive regulation of cold-induced thermogenesis, mitochondrion, lipid oxidation, and response to extracellular stimulus are activated (Figure 4C). For Ad1-*Ucp1*^Low^, pathways related to adipogenesis, response to troglitazone, and regulation of kinase activity showed more activation.

To gain additional information about the central metabolic pathways activated in the Ad1-*Ucp1*^High^ and Ad1-*Ucp1*^Low^, we analyzed the DEGs of the primary genes involved in distinct energy metabolism pathways (Figure 4D and Figure S4C). The group of genes related to TCA is preferentially activated in Ad1-*Ucp1*^High^ over Ad1-*Ucp1*^Low^. This profile was followed by the upregulation of genes involved in the following pathways: fatty acid oxidation, fatty acid transport, and glycolytic processes. Interestingly, Ad1-*Ucp1*^Low^ has a higher enrichment of triglycerides / fatty acid cycle genes than in Ad1-*Ucp1*^High^. This fact suggests that other metabolic pathways, particularly those involved in a futile cycle, could be affected (Figure 4D and Table S19). Aware of this fact, we also evaluated non-canonical or UCP1 independent pathways involved in the thermogenic program, such as glycolytic pathway, creatine metabolism genes, such as *Gamt*, *Gatm*, and *Ckmt1*, and SERCA2-pathway, such as *Arpc2*, *Adra1a*, *Atp2a2*, and *Tmlc4* (Figure S4C). Overall, there were no categorical differences in these programs when comparing Ad1-*Ucp1*^High^ and Ad1-*Ucp1*^Low^. However, concerning the glycolytic pathway, *Ppara*, *Pkm*, and *Ogdh*, particularly the former, are differentially expressed in Ad1-*Ucp1*^High^, while *Atp2a2* is equally represented in Ad1-*Ucp1*^High^ and Ad1-*Ucp1*^Low^ adipocytes. For Ad1-*Ucp1*^Low^, triglycerides/fatty acid processes and de novo lipogenesis are more enriched, with highlights for the genes; *Fasn*, *Srebf1* and *Insig1*.

Thirty-seven membrane genes were upregulated exclusively in Ad1-*Ucp1*^High^ and 14 genes upregulated in Ad1-*Ucp1*^Low^ (Figure S4D and Table S20). The top five highly expressed genes are *Slc4a4*, *Slc36a2*, *Kcnk3*, *Atp1a2*, *and Vldlr* for Ad1-*Ucp1*^High^, and *Slc27a1*, *Slc1a5*, *Slc7a10*, *Ghr*, *and Adrb3* for Ad1-*Ucp1*^Low^ (Figure 4E). The top five genes predicted to encode secreted proteins for Ad1-*Ucp1*^High^ were *Col27a1*, *Vldlr*, *Il15ra*, *Psap*, and *Ctsz*, while for Ad1-*Ucp1*^Low^ were *Ghr*, *Acvr1c*, *Lama4*, *Col15a1*, and *Retn* (Figure 4E).

Given that TFs are crucial for defining cell identity, we analyzed TF expression levels in the Ad1 subpopulation. For this analysis, we initially evaluated the TFs that are most likely involved in regulating DEGs for Ad1-*Ucp1*^High^ and Ad1-*Ucp1*^Low^ (Figure S4E,F). The former exhibited specific expression of the main canonical adipocyte TFs (such as *Parpd*, *Hnf4a*, *Ers1*, *Pparg*, *Sall4*, *Cebpd*, *Egr1*, *Nanog*, *Stat3*, *and Bhlhe40*). Protein-protein interactions (PPI) analysis demonstrated that *Arpd*, *Hnf4a*, *Ers1*, *Pparg*, *Sall4*, *Egr1*, *Nanog*, and *Bhlhe40* could interact with the targeted genes differentially expressed in *Ad2-Ucp1^High^*. For Ad1-*Ucp1*^Low^, *Parpg*, *Sall4*, *Ers1*, *Tp63*, *Ar*, and *Gata2*, the most enriched TFs and PPI analysis demonstrated that *Parpg*, *Ers1*, *Tp63*, and *Gata2* could interact with the targeted DEGs. The next step was to analyze the most highly expressed TF genes (Figure 4F), in addition to exploring the genes that are targets of these TFs (Figure S4G). As expected, the canonical adipocyte TFs, such as *Pparg*, are expressed at higher levels than the other TFs in both Ad1-*Ucp1*^High^ and Ad1-*Ucp1*^Low^, being more intense in the former. On the other hand, *Egr1* and *Ar* are more pronounced in Ad1-*Ucp1*^Low^ adipocytes. The target genes related to TFs, *Slc4a4*, *Slc25a42*, and *Pdk4*, were more pronounced in Ad1-*Ucp1*^High^ adipocytes while *Slc1a5* and *Fasn* were upregulated in Ad1-*Ucp1^L^*^ow^ compared to Ad1-*Ucp1*^High^. Finally, to show the broader applicability of the pipeline for characterizing different populations of adipocytes and establishing a relationship with their membrane markers, Figure 4G shows the intracellular and membrane markers for Ad1-*Ucp1*^High^ are *Slc36a2* and *Acadm*, while *Slc27a1* and *Fasn* correspond to Ad1-*Ucp1*^Low^. Furthermore, this tool was also efficient in rediscovering populations of beige cells previously characterized in vivo, such as glycolytic G beige adipocytes (*Eno1* and *Pkm2*) [67], that have high expression of *Eno1* and *Pkm2* in the CL-adipocytes population, while *Ckb*/*Alpl* beige cells are prominent within in the population from cold-challenge animals [68,69].

## 4. Discussion

This report presents a pipeline able to analyze existing single-nuclei transcriptome data to gain a greater understanding of the cellular composition of WAT. Here, we generate a comprehensive cellular atlas and classification of the adipocytes into five distinct subpopulations. This allowed us to delineate in vivo trajectories and determine the plasticity of individual adipocyte subpopulations from the inguinal fat pad of mice in the setting of thermogenic challenges. Cold and CL both induce UCP1+ cell populations (Ad1-*Ucp1*^High^) in addition to two other adipocyte subpopulations that show gene expression signatures distinct from each other. We also identified a new adipocyte population (Ad5) specific to CL treatment, which demonstrates enrichment for lipid turnover and de novo lipogenesis pathways, suggesting this subpopulation provides a higher energy output in a UCP1-independent fashion. In addition, the pipeline was efficient in identifying other beige adipocyte populations already established in vivo, such as glycolytic beige adipocytes. We further showed that the different adipocyte subpopulations presented specific secretome profiles, mainly composed of proteins secreted via classical and non-classical pathways (such as exosomes).

In WAT, beige remodeling can be triggered through two stimuli: Cold and CL [8,15]. Recent studies suggest that these two stimuli might induce beiging through distinct pathways and our data support those observations [8,70]. The CL-induced subpopulation showed significant oxidative phosphorylation and ATP metabolic processes, whereas cold-induced thermogenic adipocytes are more specialized for fatty acid transport. As expected, a unique mature adipocyte subpopulation (Ad1) showing a thermogenic “classic” signature, such as *Ucp1*, *Cidea*, *Dio2*, *Elovl3*, *Cpt1b*, and *Plin5*, was detected in both treatments. The thermogenic population showed to be specialized in the following pathways: fat acid oxidation, TAC, and fatty acid transport.

Interestingly, CL treatment also resulted in an additional subpopulation, Ad5. Despite not having a classic thermogenic signature, this adipocyte subpopulation showed a profile for activating genes related to glycolytic, fatty acid turnover, and, in particular, de novo lipogenesis pathways, also present at some level in thermogenic Ad1. Ad5 also showed specialization in lipid turnover pathways, suggesting this subpopulation provides a higher energy output. *Acadm*, *Cox8b*, and *Gk* are associated with both Ad1 and Ad5 subpopulations, while *Fasn*, *Acly*, and *Insig1* is more highly associated with the Ad5 subpopulation. This set of results, predominantly generated in silico, rediscovery of the ex-vivo analysis presented by Lee, et al., 2017 [71]. In this paper, Granneman and colleagues showed that CL upregulated FASN and MCAD in distinct adipocyte populations: high MCAD expression in multilocular adipocytes that co-expressed high UCP1 levels, while FASN expression occurred in paucilocular adipocytes with low UCP1 levels. These results corroborate the concept of metabolic heterogeneity as a distinct property of activated thermogenic adipocytes. However, the function of each population and the control mechanisms involved need further analysis.

Regarding the secretome of thermogenic subpopulations, Ad1 adipocytes differentially overexpressed *Nrg4*, *Col27a1*, *Psap* and *Dcn*, whereas the Ad5 subcluster over-expressed mainly *Hpsa5*, *Hsp90b1* and *Cd1d1*. Neuregulin 4 (NRG4) was previously shown as an AT-enriched secreted factor, markedly increased during brown adipocyte differentiation. *Nrg4* is downregulated in AT during rodent and human obesity. In contrast, gain-and-loss-of-function studies in mice showed it protects against diet-induced insulin resistance and hepatic steatosis [72]. Interestingly, NRG4 promotes neurite outgrowth during cold-challenge [73]. CD1d is a lipid antigen-presenting molecule for iNKT cells (invariant Natural Killer T), highly expressed in adipocytes than any other cell types of adipose tissue [74]. Adipocytes positively express CD1d molecules in lean adipose tissue, which play a crucial role in maintaining the adipose iNKT cell population. Upon HFD feeding, adipocytes present obesity-related lipid antigens via CDld molecules, which leads to iNKT cell activation and stimulates anti-inflammatory cytokine secretion from adipose iNKT cells [75]. In this sense, the adipocyte secretome prediction has shown that both NRG4 (Ad1) and CD1d (Ad5) are specific thermogenic adipocyte products since sufficient mRNA levels molecules were not detected in other cell populations from WAT.

To fully understand the potential therapeutic relevance of beige remodeling, it is crucial to characterize the overall metabolic properties of beige adipocytes in addition to their thermogenic potential. In this regard, the Ad1 subpopulation was subdivided into two subpopulations, according to the presence or absence of *Ucp1* mRNA. Interestingly, although Ad1-*Ucp1*^High^ showed a “canonical” thermogenesis genetic signature, by expressing higher DEGs corresponding to energy metabolism, particularly those related to TCA, Ad1-*Ucp1*^Low^ adipocytes showed higher triglycerides/fat acid cycle and de novo lipogenesis pathways. Also, specialization of fatty acid metabolism (oxidation and transport) and glycolytic processes are more pronounced in Ad1-*Ucp1*^High^ despite a small portion of Ad1-*Ucp1*^Low^ adipocytes having a similar profile to that found in Ad1-*Ucp1*^High^. Ad1-*Ucp1*^High^ showed, in addition to the positive regulation of thermogenesis, specialization related to response to extracellular stimulus and cellular carbohydrate metabolic process. Our analyses also showed that the UCP1 independent pathways involved in the thermogenic program, such as glycolytic, and SERCA2-pathway are equally expressed in Ad1-*Ucp1*^High^ and Ad1-*Ucp1*^Low^ adipocytes. This fact suggests that other metabolic pathways, particularly those involved in a futile cycle (lipid turnover and SERCA2), could also be involved in the thermogenic metabolism in Ad1 adipocytes in a way that does not depend on the presence or absence of UCP1. In addition to that, some elegant papers have recently provided pieces of evidence that futile cycling between creatine and phosphocreatine is part of a newly described thermogenic pathway [68,69,76]. In this regard, our analysis shows a high density of CKB (creatine kinase B) and TNAP (tissue-nonspecific alkaline phosphatase, encoded by *Alpl*) in Ad1-*Ucp1*^High^, a fact that was more evident in cold stimulated adipocytes from iWAT. Furthermore, it should be highlighted that Ad1-*Ucp1*^Low^ adipocytes make up about 75% of the adipocyte subpopulation under CL treatment, suggesting its relevance for the detailed understanding of beige remodeling.

In recent years, beige remodeling has been demonstrated through the existence of other thermogenic pathways in a UCP1-independent manner [77,78]. Even more recently, Kajimura and colleagues showed ‘glycolytic beige’ (g-beige), with significant enrichment of genes involved in glycolysis, glucose, and carbohydrate metabolism distinct from both the classical beige and brown adipose signatures [67]. The analyses presented here showed high levels of *Eno1* and *Pkm2* in Ad1-*Ucp1*^High^ cells that is prevalent in Ad1 and Ad5 adipocytes (CL-treatment). Also, as described above, these cells were enriched for genes related to glycolytic processes. This result confirms the existence of g-beige population and provides insights into the possible roles of these cells being associated with controlling thermogenesis and glucose homeostasis.

These findings motivated us to identify new markers for thermogenic adipocyte types using snRNA-seq data from digested AT samples. Although some consistent studies have presented the transcriptome profile of beige cells, either of bulk [9] or cells isolated by a reporter system (UCP1+) [50], as far as we know, this is the first analysis of the snRNA-Seq data to predict the profile of proteins associated with their respective functions. The membranome prediction analysis showed *Slc4a4* and *Adra1a* and *Slc36a2* as specific for Ad1-*Ucp1*^High^ surface markers, whereas *Slc27a1* and *Slc1a5* were more typical for Ad1-*Ucp1*^Low^. Our analyses show a very particular profile of surface proteins (plasmatic membrane), making possible additional functional analyses of those adipocyte populations. However, experimentally using ex vivo and in vivo or approaches such as sorting cells [50] and fluorescent imaging techniques must be determined.

Brown/beige adipocyte differentiation and activation of the thermogenic program are controlled by sequential actions of transcription factors (TFs), including EBF2, PRDM16, C/EBPΒ, PGC-1α, and PPARγ [79,80,81,82]. However, there is a lack of information on the transcription regulators possibly involved in the “plasticity” and/or “trans-differentiation” of white to beige adipocytes. To address this issue, we identified the most expressed set of genes, which encode TFs. Interestingly, in addition to the generic adipogenesis regulators also known to be involved in beige differentiation, such as *Prdm16*, *Pparg*, *Pgc1a*, and *Ppara*, we also showed *Clock* and *Ppara* (both up), for CL and Cold, respectively, and *Egr1* (downregulated) for both treatments. Zinc finger transcription factor EGR1 is a negative regulator of the fat beige program. Loss of *Egr1* in mice promotes browning in the absence of external stimulation and leads to increased *Ucp1* expression, which encodes the critical thermogenic mitochondrial uncoupling protein-1 [83]. Besides, during the trans-differentiation processes, the two treatments seem to activate a different set of genes along their trajectory. This may suggest that, although the endpoint is similar, the possible pathways activated may be different.

## 5. Limitations Should Be Addressed

Our paper shows descriptive evidence generated through in silico analysis from several angles, but the scope of this study lacks the appropriate in vivo models to show a mechanistic link to certain physiological functions. Future studies are needed to validate the metabolic impact of our findings concerning adipose tissue heterogeneity, plasticity, and remodeling. Moreover, secretome and membranome prediction using transcriptomic data as input also needs to be carefully evaluated considering the several mechanisms of transcriptional regulation in mammals. Our pipeline template proposes a global analysis of an adipocyte atlas; thus, there is no proper filter from contaminated droplets. The choice of a cutoff may be arbitrary. Finally, the adipocyte trajectory herein described may differ between depots and thermogenic challenges, and further studies are needed to outline and validate the trajectories in different adipocytes subpopulations.

## 6. Conclusions

In summary, we present a multidimensional reanalysis at a single-nucleus resolution, which allows the recovery of nuclei of cell types in adipose tissue. Using this tool, it was possible to show the plasticity of adipocytes, which corroborates with recent data from AT snRNAseq [35,38]. In addition to the presence of a thermogenic adipocytes subpopulation positive for UCP1 (Ad1-*Ucp1*^High^), additional transcriptome analyses allowed us to infer the presence of different secretory functions under thermogenic challenges activated by metabolic pathways, especially those related to the futile cycle. It was also possible to identify some previously characterized beige cell populations (ex vivo and in vivo). On the other hand, Ad1-*Ucp1*^Low^ seems to have a relevant role, both in energy production, through the pathways independent of UCP1, and in its secretory function. This, in turn, proved to be very specific among the heterogeneous adipocyte populations. Regarding the profile of secretory molecules, most are related to proteins that make up EMC, and the highlighted candidates are potentially secreted via classical pathways and exosomes. Understanding this functional plasticity plays an essential role in establishing the mature adipocytes’ role as a “central” cell type modulating tissue remodeling. In addition, it will provide insights into the molecular basis of metabolic adaptation in physiology and disease.

## Data Availability

The results shown here are based upon data generated by the GSE133486 and the institutional ethical review board approval is not needed.

## Supplementary Materials

The following are available online at https://www.mdpi.com/article/10.3390/cells10113073/s1, Figure S1: Overview of markers and transcriptional profile of adipose tissue cells identified by the new snRNA-Seq template. Figure S2: Transcriptome-based interactome analysis reveals Ad3-Ad4 cellular interactions. Figure S3: General analysis of thermogenic treatment in matures adipocyte subpopulation. Figure S4: General characterization of thermogenic main metabolic pathways, secretome prediction, and TF in Ad1-*Ucp1*^High^ and Ad1-*Ucp1*^Low^. Table S1: List of potential markers for each cell type from metacell analysis. Table S2: Top 20 markers for each of the 4 cell types (Adipocyte, Endothelial, Immune and Progenitor). Table S3: Enriched pathways for each of the 4 cell types (Adipocyte, Endothelial, Immune and Progenitor) against GO Biological Process, Jensen Tissues and KEGG. Table S4: Enriched pathways for each of the 4 cell types (Adipocyte, Endothelial, Immune and Progenitor) against GO Biological Process, Jensen Tissues, WikiPathways and KEGG. Table S5: Top 10 markers for each of the 5 adipocyte clusters (Ad1, Ad2, Ad3, Ad4 and Ad5). Table S6: Markers for each adipocyte cluster (Ad1, Ad2, Ad3, Ad4 and Ad5). Table S7: Enriched pathways for each of the 5 adipocyte clusters (Ad1, Ad2, Ad3, Ad4 and Ad5) against GO Biologica Process, Jensen Tissues, KEGG, Mouse Gene Atlas and WikiPathways. Table S8: Enriched pathways for each of the 5 adipocyte clusters (Ad1, Ad2, Ad3, Ad4 and Ad5) against Jensen Tissues and Mouse Gene Atlas. Table S9: Membranome and secretome prediciton in RT mature adipocyte subclusters (Ad3 and Ad4). Table S10: Markers for Cold vs. RT and CL vs. RT comparisons. Table S11: GSEA enrichment of differentially expressed genes between Cold vs. RT in adipocyte clusters using all pathways available in MSigDB. Table S12: GSEA enrichment of differentially expressed genes between CL vs. RT in adipocyte clusters using all pathways available in MSigDB. Table S13: Enriched pathways for Cold vs. RT and CL vs. RT comparisons against GO Biologica Process, Jensen Tissues, KEGG and WikiPathways. Table S14: Membranome and secretome prediciton in CL mature adipocyte subclusters (Ad1 and Ad5) and Cold-challenge subcluster Ad2. Table S15: Differential expressed genes tested as a function of different clusters used for ordering pseudotime in Cold vs. RT comparison. Table S16: Differential expressed genes tested as a function of different clusters used for ordering pseudotime in CL vs. RT comparison. Table S17: Top 10 marker genes when comparing the high and how expression of Ucp1 gene in Ad1 cluster. Table S18: All marker genes when comparing the high and how expression of Ucp1 gene in Ad1 cluster. Table S19: GSEA enrichment of differentially expressed genes between High vs. Low Ucp1 expression in Ad1 cluster using all pathways available in MSigDB. Table S20: Membranome and secretome prediciton in Ad1-*Ucp1*^High^ and Ad1-*Ucp1*^Low^. Table S21: Averaging the expression levels of the TF in each different cluster.
