# Supplementary material for: Multidimensional Single-Nuclei RNA-Seq Reconstruction of Adipose Tissue Reveals Adipocyte Plasticity Underlying Thermogenic Response
Source: Cells. 2021 Nov 8;10(11):3073. doi: 10.3390/cells10113073 (PMC8618495; doi:10.3390/cells10113073)
Supplement: Supplementary file 1 [file cells-10-03073-s001.zip › cells-1410315-Supplementary Figure Captions.pdf]

Supplementary Figure Captions

Figure S1. Overview of markers and transcriptional profile of adipose tissue cells identified by the new snRNA-Seq template.
(A) *t*-SNE plot of a dataset from Rajbhandari, P *et al.* (2019) distributed by 17 clusters.
(B) Metacell analysis classification of the "fat cake" 17 clusters in 4 cell types: adipocyte (AD), endothelial (EN), immune (IM), and progenitors (PG). Pie charts show the corresponding percentage.
(C) SCCAF plot showing the over clustering optimization.
(D) SCCAF plot showing the Cross-Validation (CV) and test for the different rounds of over clustering.
(E) Unsupervised markers for each classified cell type generated by the analysis of metacell.
(F) ORA analysis of DEGs from the four cell types identified. The intensity of the color in the dotplot indicates the enrichment significance by the combined score. Circle sizes correspond to the -log10 adjusted P-value (padj). Each dot plot represents an individual gene set related to the KEGG, WikiPathways, Jensen tissues, and GO biological process

Figure S2. Transcriptome-based interactome analysis reveals Ad3-Ad4 cellular interactions.
(A) SCCAF plot showing the over clustering optimization.
(B) SCCAF plot showing the Cross-Validation (CV) and test for the different rounds of over clustering.
(C) Violin plot of the canonical mature adipocyte markers.
(D) Volcano plot representation showing the up and down-regulated DEGs for each mature adipocyte subcluster, with the top 5 up and down-regulated DEGs labeled.
(E) ORA analysis of DEGs from the four cell types identified. The intensity of the color in the dotplot indicates the enrichment significance by the combined score. Circle sizes correspond to the -log10 adjusted P-value (padj). Each dot plot represents an individual gene set related to the Jensen tissues and Mouse Gene Atlas.
(F) Pie charts showing the percentage of genes belonging to the secretome, membranome, and other cellular compartments from Ad3 and Ad4 subclusters.
(G) Heat-scatter plot representing upregulated membranome (upper) and secretome (bottom) components identified in Ad3 and Ad4 subclusters. The color of the circles corresponds to the average logFC.
(H) Interactome representing cell-cell communication between Ad3 and Ad4 subclusters. P-values are indicated by circle size. The average expression level of interacting molecule 1 in subcluster Ad3/Ad4 and interacting molecule 2 in subcluster Ad4/Ad3 are indicated by color.

Figure S3. General analysis of thermogenic treatment in matures adipocyte subpopulation.
(A) Gene-expression heatmap of the DEGs of mature adipocyte nuclei subclusters according to each treatment (Cold, CL, and RT) compared to all others. Genes are represented in rows and cell clusters in columns.
(B) Selected top categories from ORA analysis of DEGs from mature adipocyte nuclei subclusters according to each treatment (Cold vs. RT and CL vs. RT). The intensity of the color in the dotplot indicates the enrichment significance by the combined score. Circle sizes

correspond to the -log10 adjusted P-value (padj). Gene set names are colored according to the GO biological process (purple), Jensen tissues (red), Kyoto Encyclopedia of Genes and Genomes (KEGG, blue), and WikiPathways (orange).

(C) Heat maps showing the expression of genes related to fatty acid oxidation, tricarboxylic acid cycle, fat acid transport, glycolytic process, triglyceride/fatty acid cycle, and *de novo* lipogenesis in the five mature adipocytes subclusters.

(D) Venn diagram representing the overlapping between Ad1 and Ad5 subclusters identified in the secretome, membranome, and other cellular locations.

(E) Pie charts showing the percentage of genes belonging to the secretome, membranome, and other cellular compartments from Ad1 and Ad5 (CL) and Ad2 (Cold).

(F) Heat-scatter plot representing upregulated membranome (left) and secretome (right) components identified in Ad1 and Ad5 subclusters. The color of the circles corresponds to the average logFC.

(G) Heat-scatter plot representing upregulated membranome (left) and secretome (right) components identified in Ad2 subcluster. The color of the circles corresponds to the average logFC.

(H) Interactome representing cell-cell communication between Ad1 and Ad5 subclusters. P-values are indicated by circle size. The average expression level of interacting molecule 1 in subcluster Ad1/Ad5 and interacting molecule 2 in subcluster Ad5/Ad1 is indicated by color.

(I) Monocle-generated plots presenting pseudotime ordering and differentiation trajectory of CL and RT conditions. The five monocle assigned states are shown. Green background represents the three main thermogenic genes (classic). The yellow background represents the earlier expressed genes in the trajectory. The blue background represents the later expressed genes in the trajectory. The characterized genes are DEGs throughout the trajectory.

(J) Monocle-generated plots presenting pseudotime ordering and differentiation trajectory of Cold and RT conditions. The seven monocle assigned states are shown. Green background represents the three main thermogenic genes (classic), the yellow background represents the earlier expressed genes in the trajectory, and the blue background represents the later expressed genes in the trajectory. The represented genes are DEGs throughout the trajectory.

Figure S4. General characterization of thermogenic main metabolic pathways, secretome prediction, and TF in Ad1-*Ucp1*$^{\mathbf{High}}$ and Ad1-*Ucp1*$^{\mathbf{Low}}$.

(A) Pie charts show the corresponding percentage of Ad1, Ad2 and Ad5 subclusters expressing the thermogenic genes *Ppara*, *Dio2*, *Prdm16*, *Elovl3*, and *Cox8b*.

(B) Gene-expression heatmap of all DEGs in Ad1-*Ucp1*^{High} vs. Ad1-*Ucp1*^{Low} comparison. Genes are represented in rows and cell clusters in columns.

(C) Heat maps showing the expression of genes related to glycolytic process, arginine/creatine and proline metabolism V, and SERCA2 pathways in the Ad1-*Ucp1*^{High} and Ad1-*Ucp1*^{Low}.

(D) Heat-scatter plot representing upregulated membranome (left) and secretome (right) components identified in Ad1-*UCP1*^{High} and Ad1-*UCP1*^{Low}. The color of the circles correspond to the average logFC

(E) Bar plot demonstrating the -log(p-values) of the enriched transcription factors (TFs) in Ad1-*UCP1*^{High} predicted using X2K database. Protein-Protein interactions (PPI) of TFs (green nodes) and targeted genes (grey nodes). The larger the circles, the higher the betweenness centrality value of the node. Gray lines highlight the interactions. Interactions were visualized using Cytoscape v3.7.2.

(F) Bar plot demonstrating the -log(p-values) of the enriched transcription factors (TFs) in Ad1-*UCP1*^{Low} predicted using the X2K database. Protein-Protein interactions (PPI) of TFs (green nodes) and targeted genes (grey nodes). The larger the circles, the higher the betweenness centrality value of the node. Gray lines highlight the interactions. Interactions were visualized using Cytoscape v3.7.2.

(G) Heatmap showing the average expression for 42 gene targets from 7 main TFs in Ad1-*UCP1*^{High} cells (green background) and 13 gene targets from 5 main TFs in Ad1-*UCP1*^{Low} cells (yellow background). These targets and TFs are related to the DEGs from each comparison through the Transcription Factor Enrichment Analysis (TFEA) using the X2K database.
