# Supplementary material for: Multidimensional Single-Nuclei RNA-Seq Reconstruction of Adipose Tissue Reveals Adipocyte Plasticity Underlying Thermogenic Response
Source: Cells. 2021 Nov 8;10(11):3073. doi: 10.3390/cells10113073 (PMC8618495; doi:10.3390/cells10113073)

Supplementary Figure S1

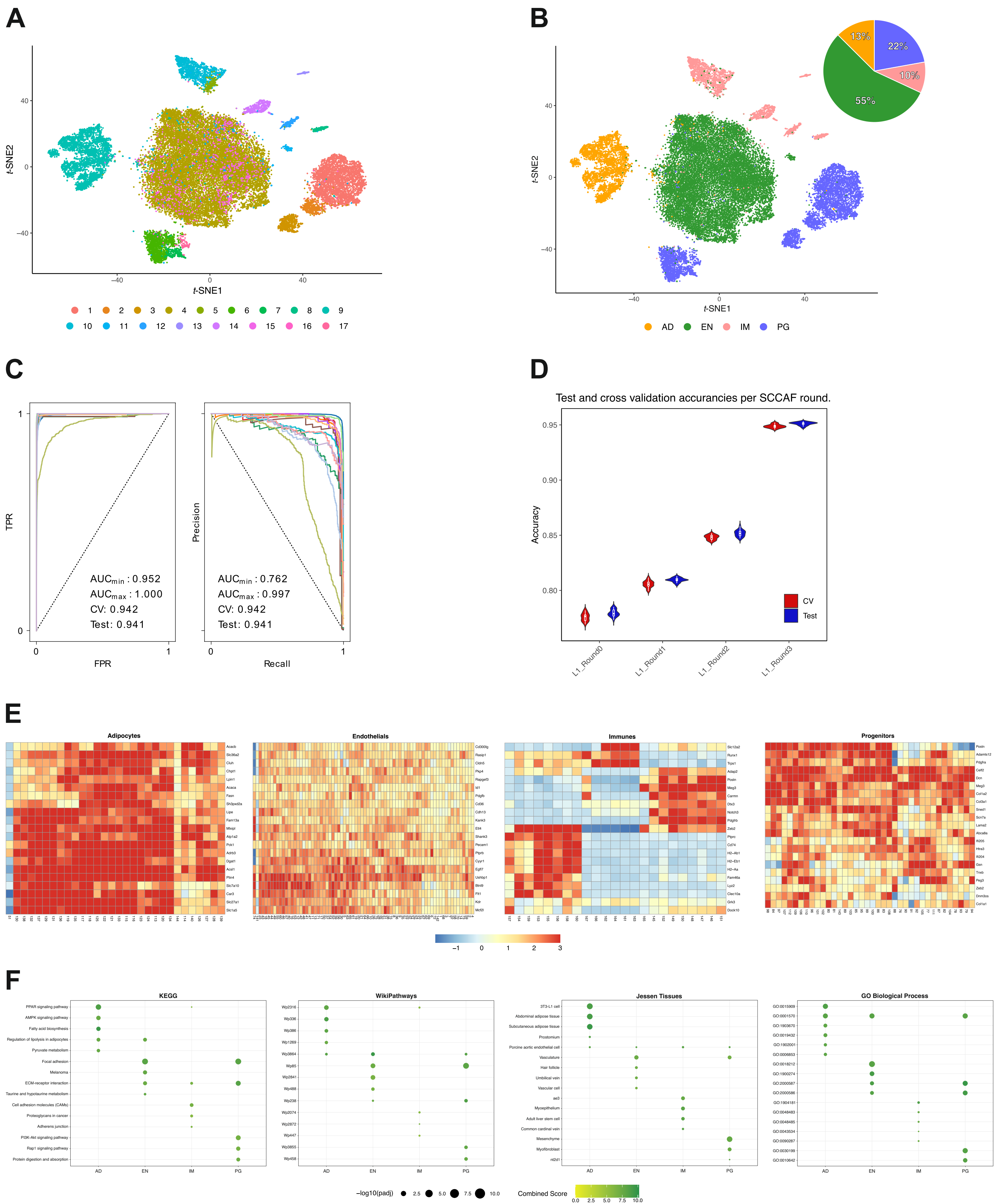

Supplementary Figure S2

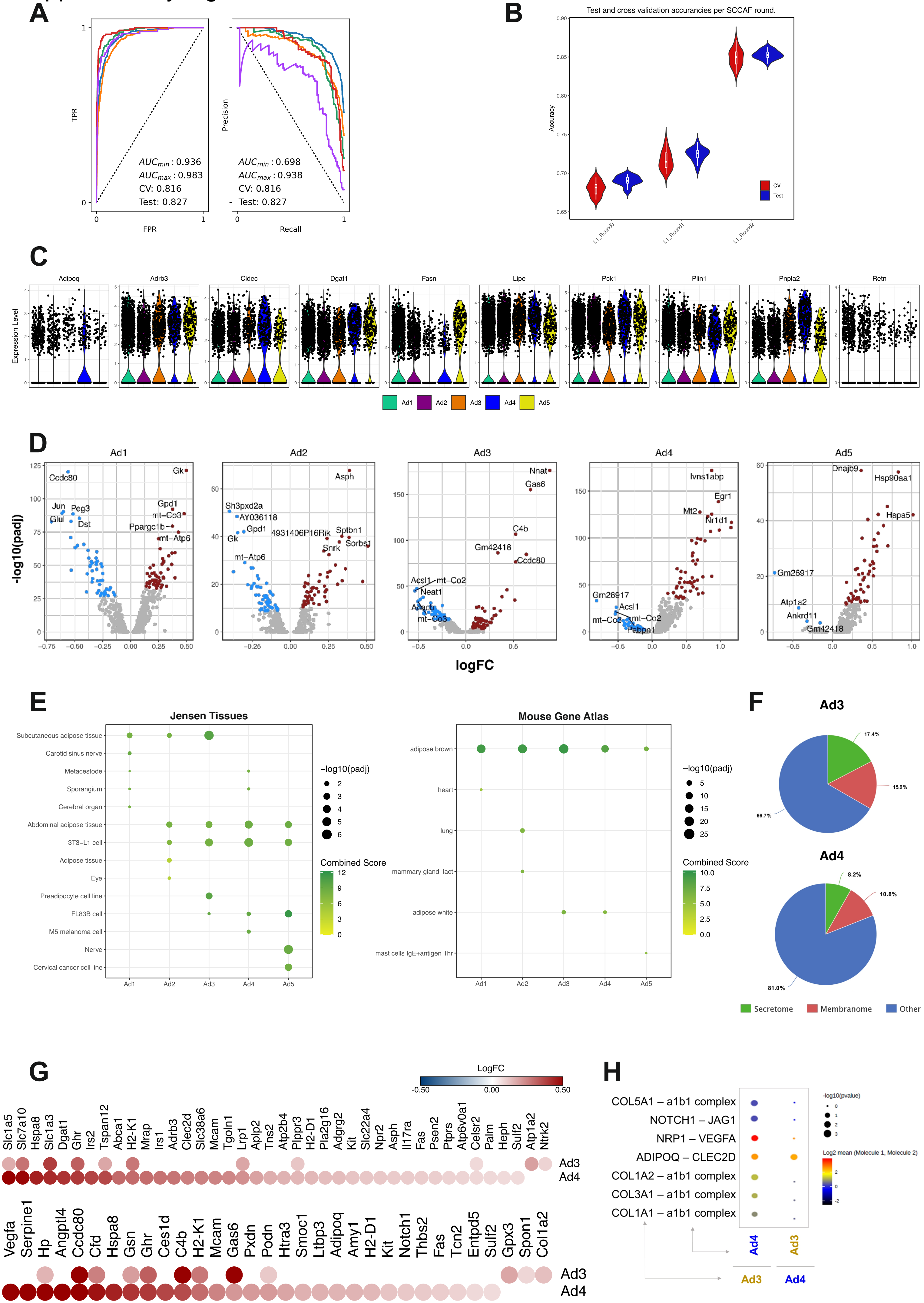

Supplementary Figure S3

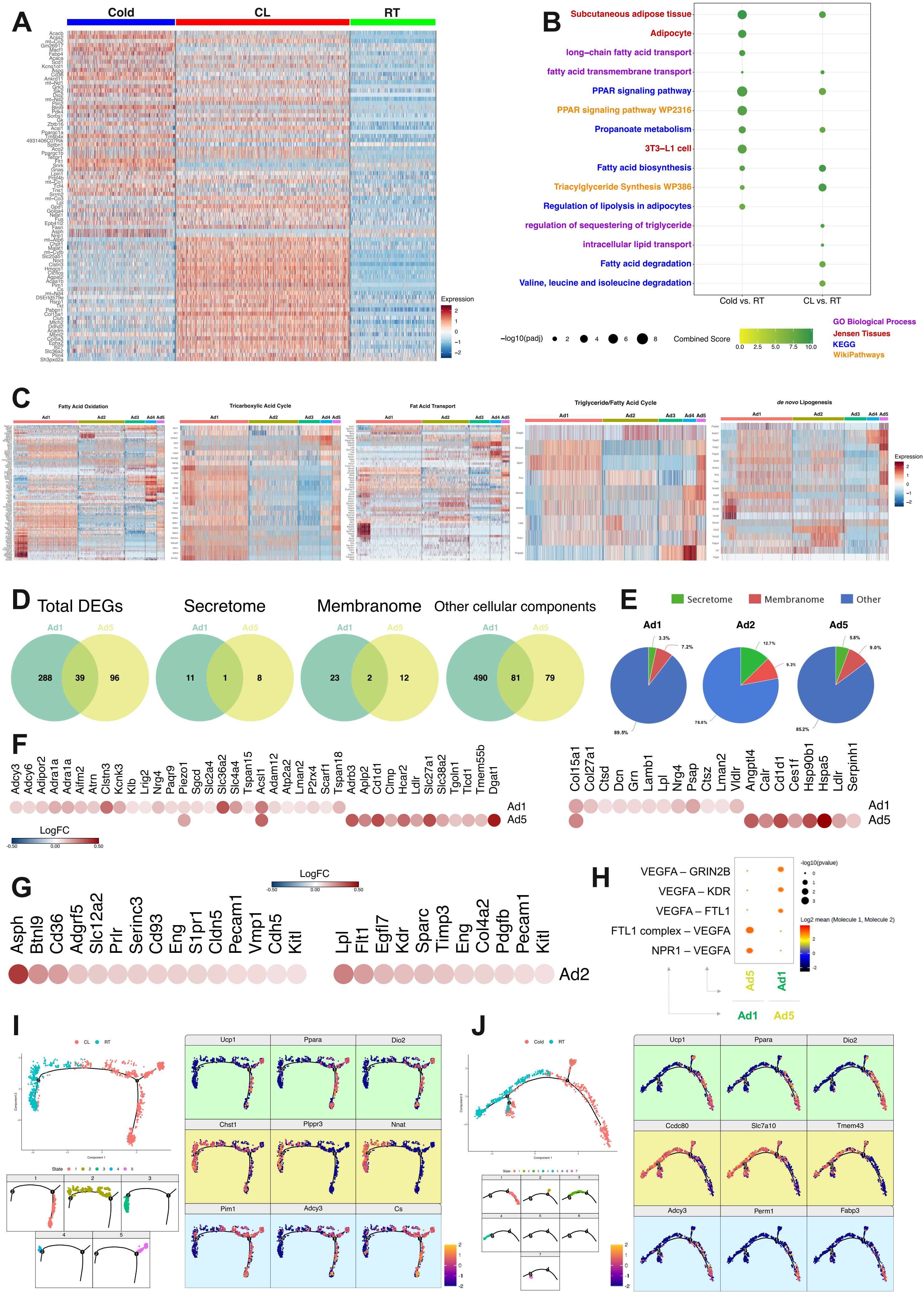

Supplementary Figure S4

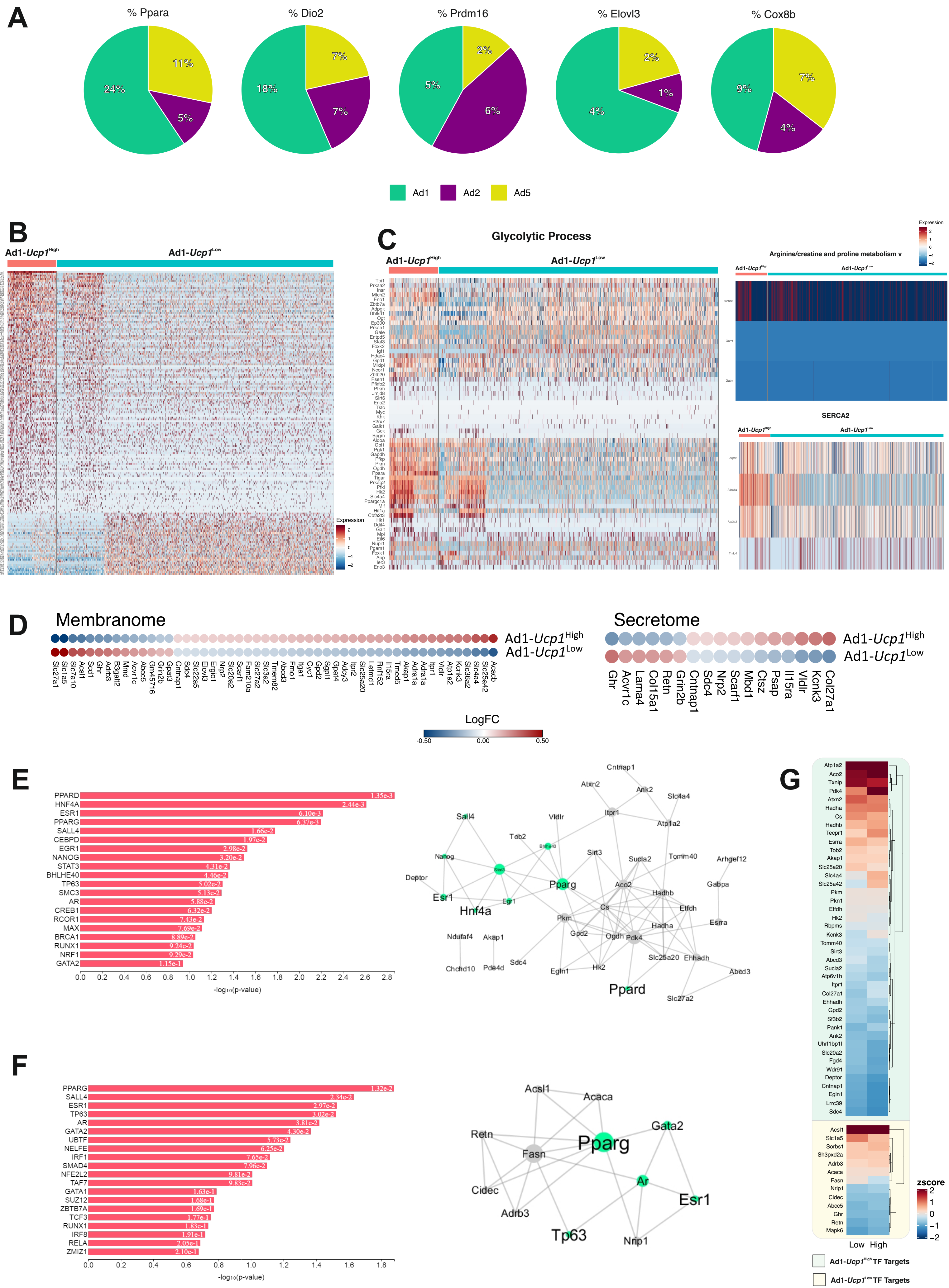

Supplement: Supplementary file 1 [file cells-10-03073-s001.zip › cells-1410315-supplementary figure resubmitted.pdf]
